# Sea-level rise and extreme Indian Ocean Dipole explain mangrove dieback in the Maldives

**DOI:** 10.1038/s41598-024-73776-z

**Published:** 2024-11-12

**Authors:** Lucy Carruthers, Vasile Ersek, Damien Maher, Christian Sanders, Douglas Tait, Juliano Soares, Matthew Floyd, Aminath Shaha Hashim, Stephanie Helber, Mark Garnett, Holly East, Jamie A. Johnson, Gheorghe Ponta, James Z. Sippo

**Affiliations:** 1 Department of Geography and Environmental Sciences, Northumbria University, Newcastle upon Tyne, NE1 8ST UK; 2 Department of Coastal Studies, Coastal Studies Institute, East Carolina University, Wanchese, NC 27981 USA; 3 Faculty of Science and Engineering, Southern Cross University, Lismore, 2480 Australia; 4 National Marine Science Centre, Southern Cross University, Coffs Harbour, 2450 Australia; 5 Universidade Federal Fluminense, Niterói, 24.020-141 Brazil; 6 Maldives Resilient Reefs, Blue Marine Foundation, 20285 Malé, Maldives; 7 NEIF Radiocarbon Laboratory, Scottish Universities Environmental Research Centre, East Kilbride, G75 0QF UK; 8 Geological Survey of Alabama, Groundwater Assessment Program, Tuscaloosa, 35401 USA

**Keywords:** Biogeochemistry, Environmental sciences

## Abstract

Mangrove forests enhance Small Island Developing States’ resilience to climate change, yet in 2020, a mangrove dieback impacted ~ 25% of mangrove-containing islands in the Maldives. Using remote sensing, dendrology and sediment geochemistry, we document a significant decrease in mangrove health post-2020 (NDVI: 0.75 ± 0.09) compared to pre-2020 (0.85 ± 0.04; P < 0.0001). Dead trees showed reduced stomatal conductance (*δ*^13^C: − 26.21 ± 0.11 ‰) relative to living ones (− 27.66 ± 0.14 ‰), indicating salinity stress. Critically, sea-level rise (30.50 ± 23.30 mm/year) outpaced mangrove sediment accretion (6.40 ± 0.69 mm/year) five-fold between 2017 and 2020. We attribute this dieback to salinity stress driven by record-high sea levels in 2020, linked to an extreme positive Indian Ocean Dipole event. These findings reveal the vulnerability of mangrove ecosystems to rapid sea-level rise and highlights the urgent need for adaptive conservation strategies in Small Island Developing States.

## Introduction

Mangroves are highly productive ecosystems that grow in regularly inundated saline sediments along the intertidal zone in tropical and subtropical coastlines^1^. Mangrove forests provide a wide range of valuable ecosystem goods and services, especially for inhabitants of Small Island Developing States (SIDS), who rely heavily on mangroves to support their subsistence and well-being^2^. For these communities, mangroves act as a source of energy, construction materials for housing, and provide medicinal resources, including Jemuju and Nipa fruit^2^. The marine biodiversity supported by mangroves also enhances food security and livelihoods through the provision of important protein sources, such as crabs, prawns, and fish^3^. Notably, mangrove forests play a crucial role in enhancing the physical resilience of these nations to the impacts of climate change by providing a natural barrier to coastal erosion, and by protecting shorelines from natural hazards (e.g., typhoons or cyclones) and relative sea-level rise (RSLR)^4^.

By 2100, global sea level is projected to rise between 0.43 m and 0.83 m under low (Relative Concentration Pathway, RCP, 2.6) and high (RCP 8.5) CO_2_ emission scenarios, respectively^5^. Rapidly rising sea level threatens mangrove resilience^6^ with mangrove retreat considered likely when relative sea level rise exceeds 4 mm/year and highly likely above 7 mm/year^7^. In Small Island Developing States, mangroves are often unable to migrate landward due to limited land size, coastal development and low sediment supply^8^. Therefore, to keep pace with rising sea levels, mangroves must increase their surface elevation sediment accretion^9^. However, comparisons of sediment accretion rates versus local average sea-level rise suggest that mangroves have been unable to keep pace with rising sea levels on low-lying islands in both the Caribbean and Pacific regions^8,10,11^. Failure to adjust to sea-level rise results in the permanent submergence of the mangrove sediment surface, altering sediment biogeochemistry, which may cause substrate collapse and tree mortality^12,13^.

Extreme climatic events represent an emerging threat to mangroves globally^14^. These events can be driven by ocean-atmospheric processes, such as the El Niño-Southern Oscillation (ENSO) and the Indian Ocean Dipole (IOD) which can lead to significant changes in sea level, rainfall and temperature^15^. When superimposed on underlying stresses of climate change, extreme climatic events can push ecosystems beyond their tipping points^7^. This was seen in Australia in 2015 and 2016 as the ENSO-related decrease in sea level (of up to 20 cm) and a prolonged period of drought, resulted in multiple large scale mangrove diebacks^16–19^. Moreover, elevated sea levels associated with extreme climatic events can accelerate the regional threat posed by sea-level rise, increasing mangrove vulnerability^16^. The scale of mangrove diebacks associated with extreme events is a function of exposure, intensity, species composition, and the forests’ geomorphic setting^20^. As the magnitude and frequency of extreme events increase with climate change, extreme sea level oscillations are generated with little recovery time for coastal ecosystems which is predicted to drive a net decrease in population size^14^.

Small Island Developing States are highly dependent on mangrove ecosystem services, therefore, mangrove loss disproportionately affects the inhabitants of these nations compared to high-income countries^21^. Depletion of mangrove forests will directly impact local economies, livelihoods and food security as mangroves contribute considerably to near-shore fisheries such as bivalve, shrimp and crab fisheries^22–24^. For instance, Guinea-Bissau in West Africa has the greatest percentage (96%) of small-scale fishers fishing in mangroves, while in the Caribbean, the biomass of commercially important species more than doubles when their habitat is connected to mangrove forests^24^. As the protective barriers provided by mangroves are lost, the susceptibility of Small Island Developing States to natural hazards and relative sea level rise will intensify. Increased shoreline vulnerability is especially concerning considering that these nations are already vulnerable to the effects of climate change. For example, six out of the 10 countries most at risk from a one-in-250-year cyclone are Small Island Developing States^25^. Further, Antigua and Barbuda, in the Caribbean, have the highest relative average annual losses globally of gross domestic product (GDP) associated to cyclone wind while the Bahamas and Dominica have the highest relative risk with respect to storm surges^25^. If substantial socio-economic damage occurs because of mangrove loss, economic recovery may be challenging as Small Island Developing States include some of the world’s least developed countries^26^.

As the world’s lowest lying nation (< 1.5 m above mean sea level)^27^, mangrove forests in the Maldives experience the compounding effects of high rates of sea-level rise (up to 4.24 mm/yr)^28^ and limited land area (~ 300 km^2^)^29^, and are therefore likely to be highly susceptible to the adverse impacts of sea-level rise. In 2020, a widespread mangrove dieback occurred in the Maldives. Analysis of satellite images suggests the dieback potentially affected up to approximately 26% of islands containing mangrove forests. Preceding the mortality event, there had been no large storm events or tropical cyclones, which are typically associated with mangrove mortality^30^. Additionally, as the dieback occurred on both inhabited and uninhabited islands, local anthropogenic drivers were unlikely. In the absence of these drivers, we hypothesise that sea level-rise was the most likely cause of mangrove dieback in the Maldives in the 2020 event. Here, we test our hypothesis by combining multiple lines of evidence from analyses of sea level, climate data and remote sensing with field observations of sediment geochemistry and dendrology. Given the nation’s extremely low elevation, this dieback serves as a precursory warning to mangroves in low-lying islands globally, by offering insights into the impacts of future sea level rise.

## Evidence of sea-level rise driving mangrove dieback in the Maldives

### Study area

**Fig. 1:**
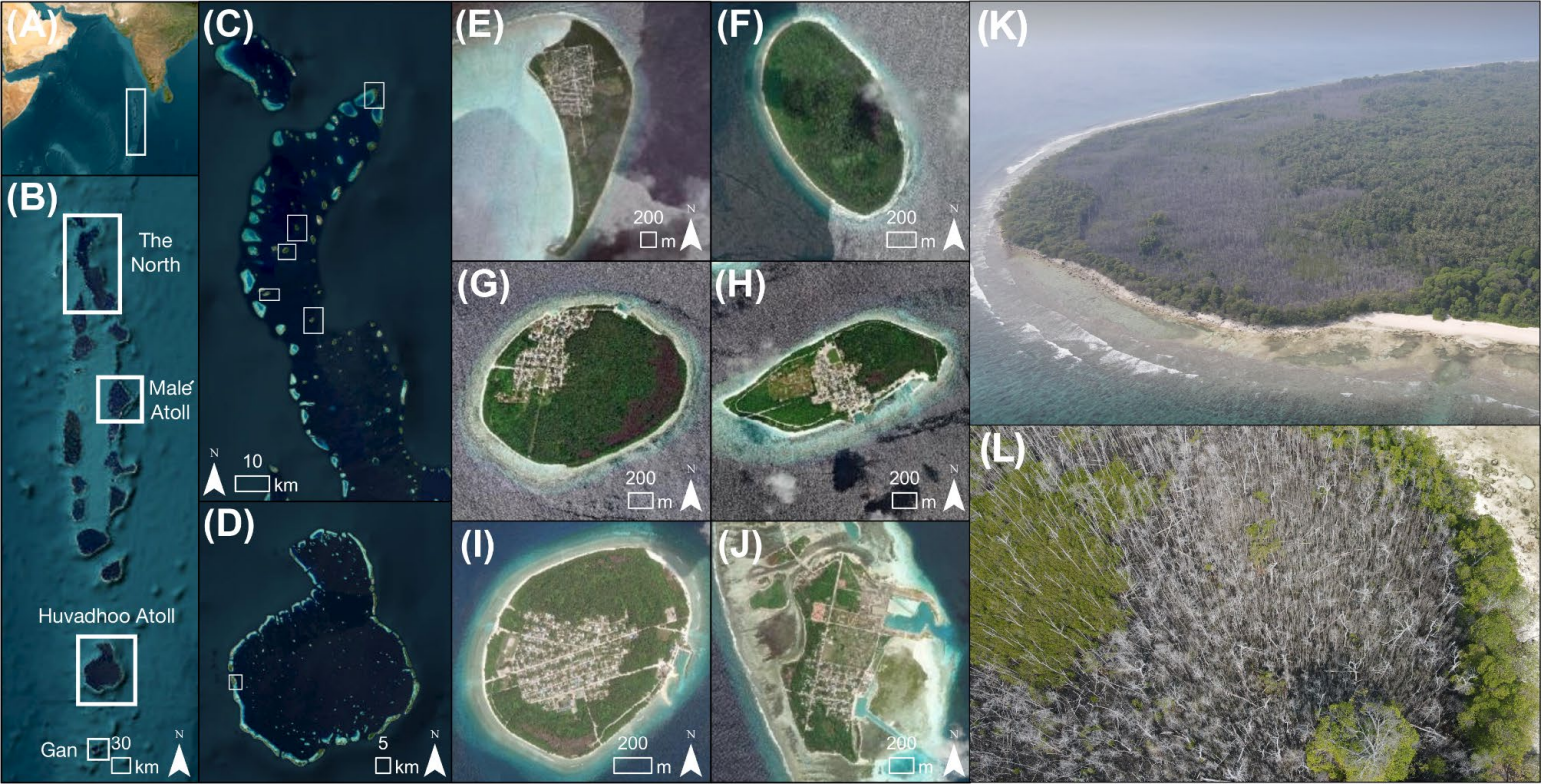
The Maldives. **(A)** The location of the Maldives within the Indian Ocean, **(B)** The Maldives Archipelago, **(C)** The five study islands in the North, **(D)** Location of the study island in Huvadhoo Atoll, in the southern region of the Maldives, **(E)** Remote sensing sites (HA. Kelaa, HDh. Keylakunu, HDh. Neykurendhoo, Sh. Goidhoo, Sh. Feydhoo, GDh. Hoandedhdhoo); and **(F)** GDh. Hoandedhdhoo Island as the field site for dendrology and sediment geochemistry. (Imagery source: ESRI, 2023 and Lucy Carruthers. Software used to generate maps; ArcGIS Pro version 2.9.0, https://www.esri.com/en-us/arcgis/products/arcgis-pro).

The Maldives is a 900 km long archipelago situated in the central Indian Ocean, extending from 6°57′ N to 0°34′ S (Fig. 1). The Maldives archipelago comprises of 1,190 coral reef islands formed from unconsolidated Holocene sediment^31^. Sea-level history from radiometrically dated reef cores suggest that the Maldives experienced a middle to late Holocene highstand between 2000–4000 yr B.P (0.50 ± 1 m above current sea level at highstand) after which sea level fell to its current position^32^. Mangrove forests in the Maldives are situated on carbonate coral reef islands where sediment is formed from mangrove-based peat, rather than terrigenous sediment, which dominates other geomorphic settings such as deltaic and estuarine forests^33^. Mangroves are present on at least 150 islands across the Maldives^34^ and typically develop in two different geomorphic settings: (1) open bay mangroves which are connected to the sea by open channels; and (2) basin mangrove forests where there is little connection to seawater and are, thus, more hydrologically isolated^34,35^. A total of 15 mangrove species are present within the Maldives, four of which exhibit low tolerance to salt, with a further five exhibiting mid-tolerance to salt^36,37^. The dieback mainly affected the species *Bruguiera cylindrica* which has a low salinity tolerance and lacks salt glands^36^. Further, the majority of impacted mangrove forest areas were those within basins, where tidal flushing is limited, and the elevation is lower.

We focused on six islands in the Maldives (Table 1). These six sites have basin mangrove forests which are situated parallel to the shoreline and behind carbonate banks^33^. Islands experience varying amounts of annual precipitation and rates of sea level rise with northern islands experiencing 1779 mm^38^ and 4.12 ± 1.22 mm/yr^39^, respectively, while southern islands experience 2218 mm ^38^ and 3.07 ± 0.97 mm/yr^39^, respectively. All sites have semidiurnal tides, with a tidal range of up to 1.4 m^40^.

**Table 1:** Characteristics of islands studied.

Island	Lat	Long	Location	Location within the atoll	Island type	Population size	Forest geomorphology	Species effected
Kelaa	6°57′21ʺN	73°12′51ʺE	North	Rim	Inhabited	1115	Basin	*B. cylindrica*
Keylakunu	6°36′08ʺN	73°00′34ʺE	North	Lagoon	Uninhabited	0	Basin	*B. cylindrica*
Neykurendhoo	6°32′33ʺN	72°58′48ʺE	North	Lagoon	Inhabited	641	Basin	*B. cylindrica*
Goidhoo	6°25′52ʺN	72°56′04ʺE	North	Lagoon	Inhabited	592	Basin	*B. cylindrica*
Feydhoo	6°21′43ʺN	73°03′0.5ʺE	North	Lagoon	Inhabited	854	Basin	*B. cylindrica*
Hoandedhdhoo	0°26′44ʺN	73°00′14ʺE	South	Rim	Inhabited	885	Basin	*B. cylindrica*

### Analyses of climatic conditions

**Fig. 2:**
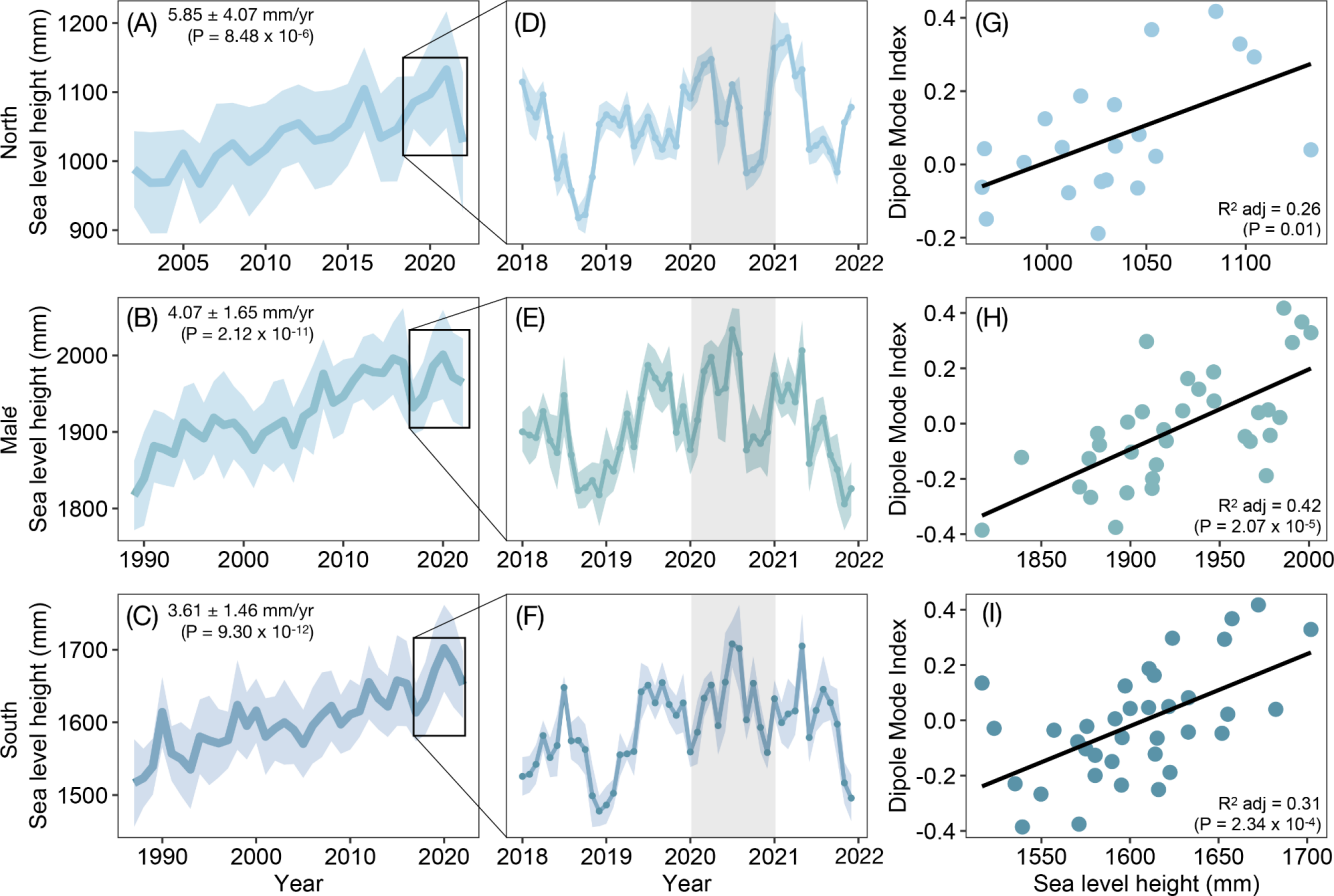
Sea level trends in the Maldives. Tide gauge records showing annual average sea level height (mm) (above datum) and rates for **(A)** HDh. Hanimaadhoo (2002 to 2022, UHSLC ID:117), **(B)** K. Malé (1989 to 2022, UHSLC ID: 108), **(C)** S. Gan (1987 to 2022, UHSLC: 109). Panels **D** to **F** show monthly averages from 2018 to 2022. Shaded areas denote standard deviation and grey vertical bars indicate the year of the dieback. Panels **G** to **I** illustrate the relationship between sea level height and the Western Indian Ocean Dipole Mode Index (DMI).

The 2020 dieback event occurred when sea level reached its highest point on tide gauge records (Fig. 2). This extreme oscillation in mean sea level coincided with an intense positive phase in the Indian Ocean Dipole. The Indian Ocean Dipole is a sea surface temperature phenomenon that can induce climate extremes for countries within the Indian-Ocean^41^. A positive Indian Ocean Dipole event begins in austral autumn–winter and peaks during austral spring. In the Western Indian Ocean, south-easterly winds bring warmer waters resulting in increased sea surface temperatures and convection^41^. During a negative Indian Ocean Dipole phase, the reverse is observed.

The 2019 to 2020 positive Indian Ocean Dipole event stands as the second strongest event in the instrumental record^42^. Initial indications of the Indian Ocean Dipole emerged with the onset of south-easterly winds in southern Maldives (S. Gan) in July 2019, deviating from the typical prevailing westerly winds, and gradually extending northward in the following months. In addition to an increased sea level, 2020 marked a record-breaking year for air temperature and sea surface temperature (Supplementary Fig. 1 and 2). We found a significant relationship between sea surface (P < 0.001, adjusted R^2^ = 0.75) and air temperature (North: P < 0.0001, adjusted R^2^ = 0.67, South: P < 0.01, adjusted R^2^ = 0.57) in connection with the Dipole Mode Index whereby a positive event produced a rise in both air and sea surface temperature (Supplementary Fig. 1 and 2). Because of the thermosteric effect, a significant positive correlation (North: P < 0.05, adjusted R^2^ = 0.26, Malé: P < 0.0001, adjusted R^2^ = 0.42, South: P < 0.001, adjusted R^2^ = 0.31) was found between sea level and the Dipole Mode Index throughout the Maldives (Fig. 2D-F). However, while a positive event brings anomalously heavy rain to the Western Indian Ocean^43^, no significant (North: P > 0.05, South: P > 0.05) relationship was found between rainfall and the Indian Ocean Dipole in the Maldives^44^ (Supplementary Fig. 3).

### Remote sensing

**Fig. 3:**
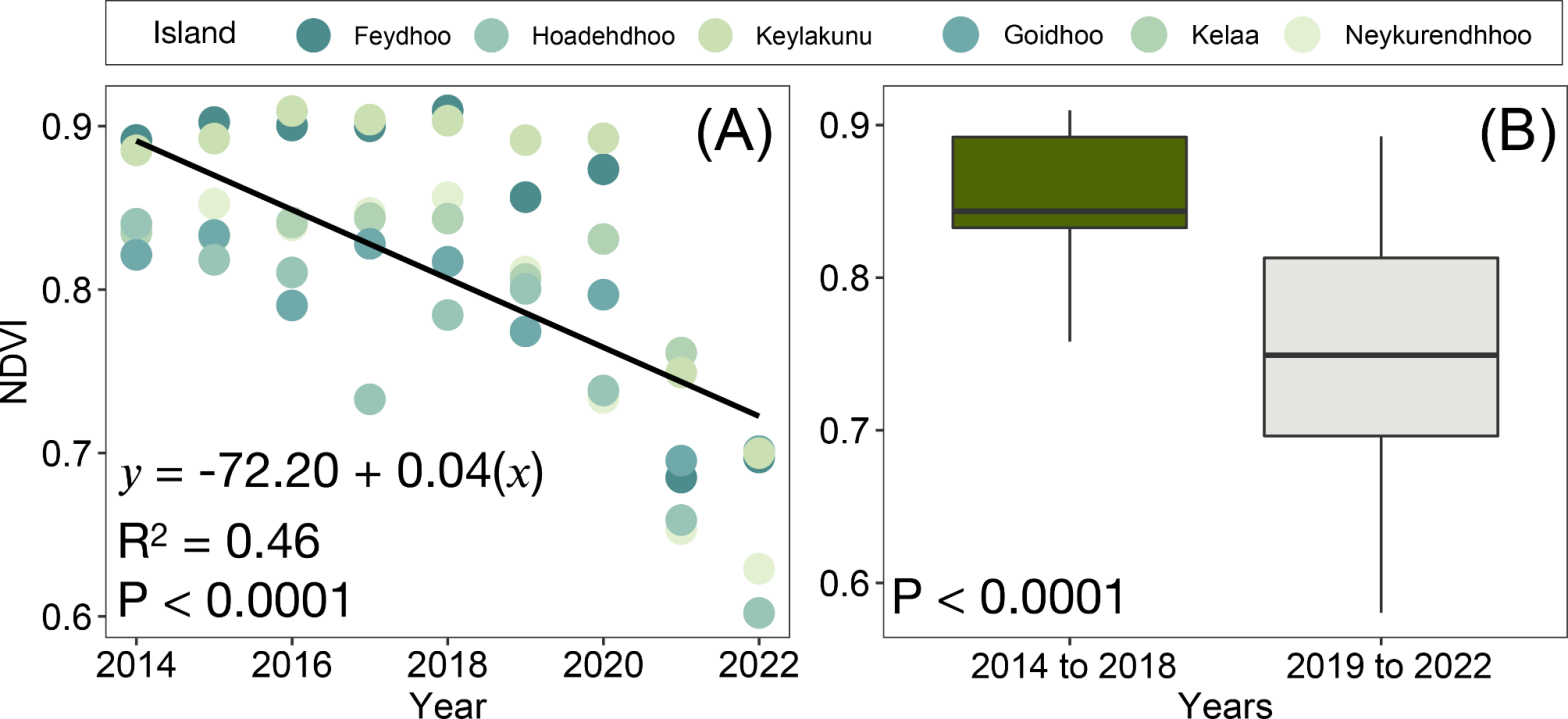
Mangrove health through time. **(A)** Temporal trends in annual NDVI for all study islands from 2014 to 2022; and **(B)** The difference in annual NDVI for all study islands between 2014 to 2018 and 2019 to 2022.

Normalised difference vegetation index (NDVI) measures the photosynthetic activity in vegetation and can thus be used to monitor spatiotemporal changes in mangrove health in response to climatic or human disturbances^45^. Examining temporal trends in mangrove health through NDVI analysis revealed a significant decrease (P < 0.001, adjusted R^2^ = 0.46) in annual average NDVI across the study period for all islands (Fig. 3A, Supplementary Table 1). Further, annual average NDVI for all study islands was significantly (t = 5.15, df = 28.89, P < 0.0001) higher before (0.85 ± 0.04) the 2019 to 2020 event compared to afterwards (0.75 ± 0.09) (Fig. 3B). Percent area loss on Neykurendhoo and Hoandedhdhoo (as of 2024) revealed losses of up to 27.38% and 53.39%, respectively.

**Fig. 4:**
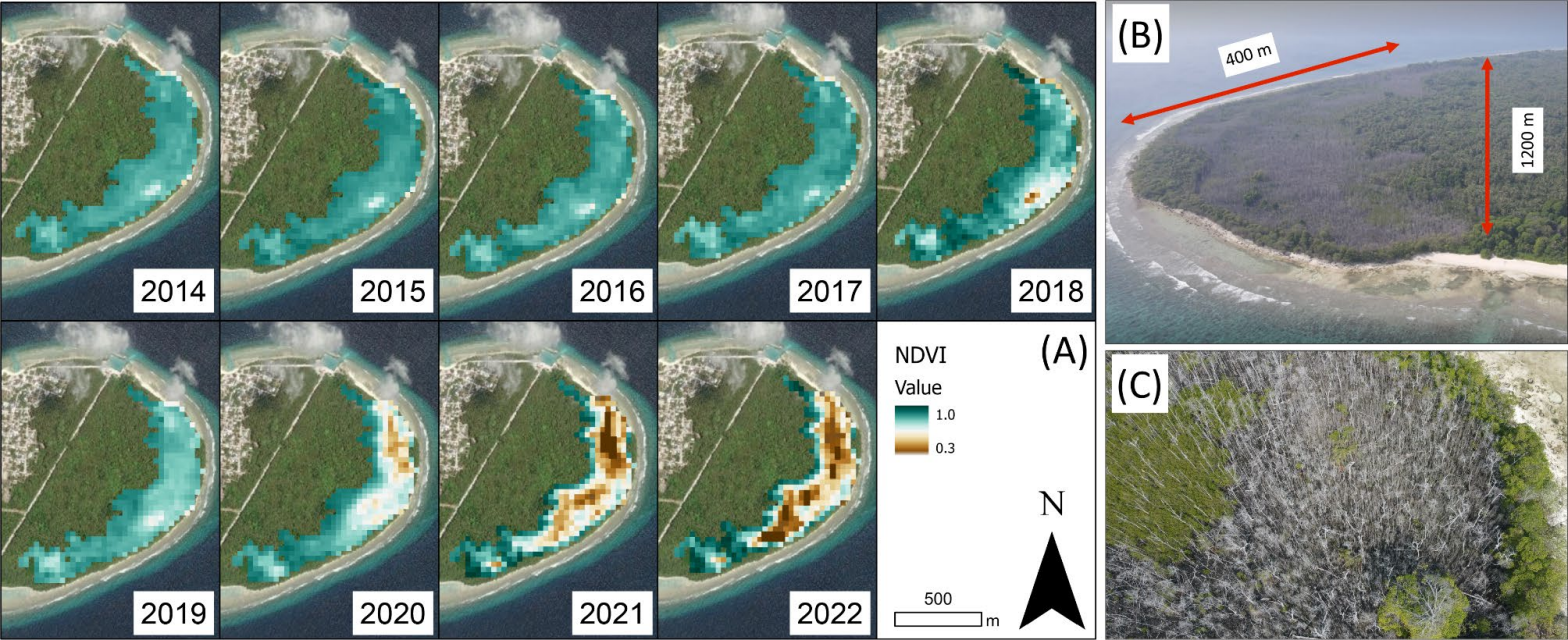
Mangrove health through time. (**A)** Temporal trends in NDVI on Neykurendhoo (Imagery source: ESRI, 2023) Software used to generate maps; ArcGIS Pro version 2.9.0, https://www.esri.com/en-us/arcgis/products/arcgis-pro); and **(B-C)** Oblique aerial drone image of mangrove dieback extent on Neykurendhoo in 2023 (Imagery source: Maldives Resilient Reefs).

No significant (North: P > 0.05, South: P > 0.05) relationship was found between annual average NDVI and annual average sea level height (Supplementary Fig. 4A-B). However, a significant negative correlation between NDVI and sea level height (North: P < 0.05, adjusted R^2^ = 0.36, South: P < 0.05, adjusted R^2^ = 0.58) was found throughout the Maldives when a one-year time lag was applied (Supplementary Fig. 4C-D). This negative correlation suggests that the mangrove forest may have been initially resilient to perturbations in sea level change, although this resilience declined with sustained inundation. The spatial distribution of the mangrove forest dieback was similar for all islands (Fig. 4 and Supplementary Fig. 5). Dieback areas were first observed in 2020 in the centre of low-lying basins and gradually spread outwards over the subsequent years (Fig. 4). This dieback pattern could be indicative of ponding (or flooding) of sea water in the lower elevation area of the depression due to the extreme rise in sea level. Limited tidal flushing and drainage within the mangrove basin may have further increased the risk of mangroves to hyper-salinization^35^.

### Dendrology and sediment geochemistry

The isotopic fractionation of carbon in mangroves is related to the ratio of intercellular (C_i_) to ambient (C_a_) partial pressure of CO_2_^46^. Consequently, fluctuations in *δ*^13^C may indicate shifts in the C_i_/C_a_ ratio due to environmental changes, such as alterations in salinity^46^. Mangroves exhibit a similar response to both increased salinity and droughts, by reducing their stomatal conductance and intercellular CO_2_ concentration^47^. This reduction leads to an increase in intrinsic water use-efficiency (iWUE), which is the ratio of photosynthetic activity over stomatal conductance, and *δ*^13^C^46^.

**Fig. 5:**
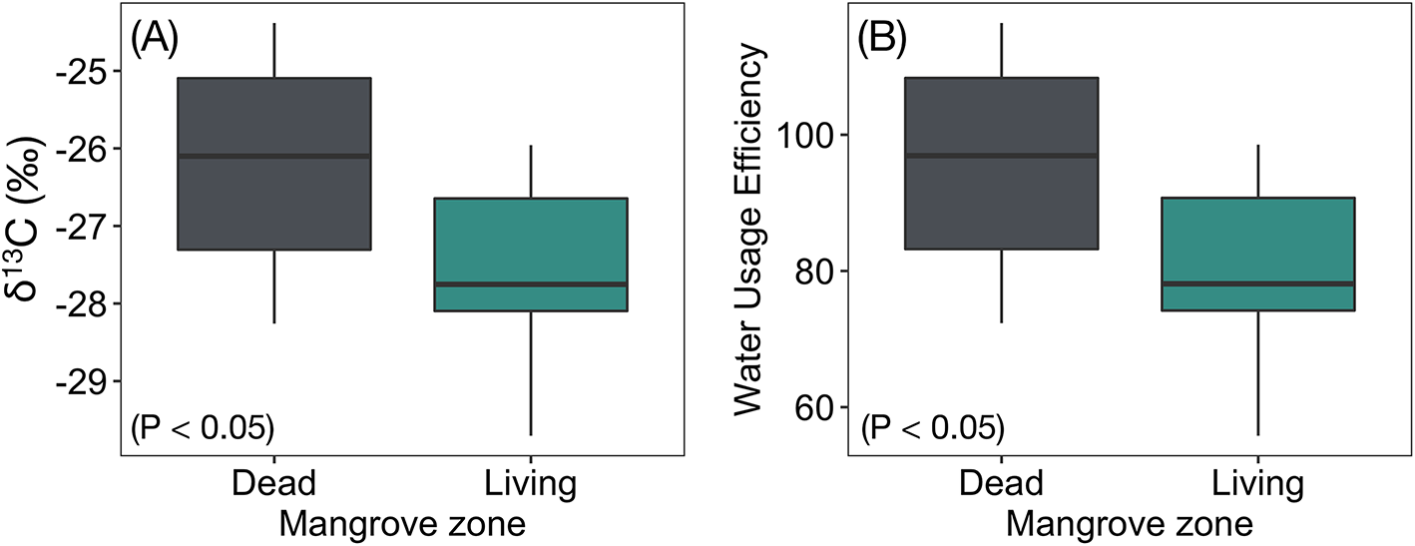
Differences in mangrove wood. The difference in **(A)** δ^13^C (‰) (P < 0.05); and **(B)** Intrinsic water use-efficiency (iWUE) between dead and living zones (P < 0.05).

Analysis of average *δ*^13^C in mangrove wood from the Maldives indicated signs of salinity stress as *δ*^13^C was significantly (t = 2.60, df = 18.10, P < 0.05) more enriched in the dead zone (− 26.21 ± 0.11 ‰) compared to the living (− 27.66 ± 0.14 ‰; Fig. 5). Further, average intrinsic water use efficiency was significantly higher (t = 2.60, df = 18.03, P < 0.05) in the dead zone (95.70 ± 1.26) compared to the living (79.11 ± 1.56).

While mangroves mainly extract porewater, groundwater can be utilised as an alternative water source^48^. On the coral reef islands of the Maldives, fresh groundwater exists as a shallow lens that floats upon denser seawater which is considered extremely vulnerable to salinization due to sea level rise, consequently limiting freshwater availability^31^. Amongst the many implications of hyper salinization, salinity stress has been shown to lead to reduced plant and root productivity as growth rates decrease^9^.

As a result of salinity stress, declining root production can lead to sediment vertical accretion deficits, increasing the risk of soil submergence and peat collapse^12,13^. This is crucial for low-lying island organogenic mangroves in the Maldives, where root production heavily influences elevation, and sea level rise is unlikely to decrease soil organic matter decay rates^49^. Comparisons of sediment accretion (mm/year) and average sea-level rise (mm/year) on Hoandedhdhoo revealed that mangroves were keeping pace with sea level rise from 1999 to 2017 (Supplementary Table 2). Between 1999 to 2017, average rates of mangrove sediment accretion were 5.39 ± 0.97 mm/year compared to 3.09 ± 3.16 mm/year for sea-level rise. Further, accretion rates accelerated from 2.8 mm/year in 1960 to 7.1 mm/year in 2017, suggesting that sea-level rise can accelerate sediment accretion rates^9,50^. Since terrigenous sediment supply is limited in the Maldives, surface accretion is most likely caused by the production of soil organic matter from vegetation such as litterfall, benthic mats and root production^9^.

On Hoandedhdhoo, mangrove sediment accretion was greater compared to those located on carbonate platforms elsewhere (e.g. Florida, USA^51–53^ and the Yucatán Peninsula, Mexico^51^. In Florida and Mexico, average 10-year (2.5 ± 0.5 to 4.8 ± 1.0 mm/year) and 50-year (2.0 ± 0.5 to 3.7 ± 0.7 mm/year) mangrove sediment accretion rates were markedly lower compared to those on Hoandedhdhoo (6.1 ± 1.4 mm/year and 4.7 ± 1.7 mm/year, respectively). The higher sediment accretion rates on Hoandedhdhoo may be driven by island geomorphology, as mangroves on Hoandedhdhoo are situated in a basin, which limits tidal flushing and the lateral export of organic matter to coastal waters^9^. Moreover, sand ridges which are formed by sediment deposition, run parallel to the mangrove basin and can restrict the outwelling of organic matter.

**Fig. 6:**
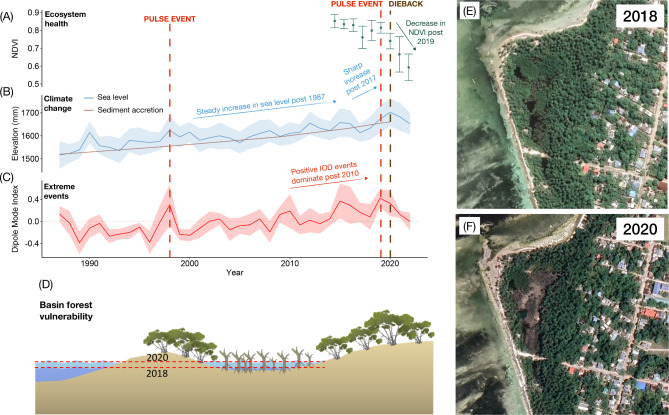
Modelling mangrove dieback on Hoadehdhoo Island. The press-pulse framework adapted from Harris et al.^14^ showing changes in mangrove health **(A)** due to climate change **(B)** and extreme events **(C)**. Dashed red lines indicate the 1997 and 2019 extreme positive Indian Ocean Dipole events. Mangrove threshold reached in 2020 when sea level reached its highest point on tide gauge records (indicated by dashed brown line). **(D)** Basin forest vulnerability due to extreme shifts in sea level rise. Satellite imagery (Google Earth Pro version 7.3.6 https://www.google.com/earth) for visualization of before **(E)** and after **(F)** the dieback.

Despite the high accretion rates we observed, mangroves in the Maldives were unable to vertically adjust their sediment surface elevation in response to the Indian Ocean Dipole-driven extreme sea level rates from 2017 to 2020 (Fig. 6). During this period, average rates of accretion were 6.40 ± 0.69 mm/year while sea level was 5 times higher (30.50 ± 23.30 mm/year). Adding to this, there was a corresponding deacceleration in sediment accretion from 7.1 mm/year in 2017 to 5.7 mm/year in 2020. Decreased rates of mangrove sediment accretion are likely a consequence of reduced growth rates and root production associated with physiological salinity stress from increased sea level. Indeed, this short-term record may indicate future trends as climate change and extreme climatic events continue to intensify^44^.

Collectively, the datasets presented in this study provide multiple lines of evidence that an extreme increase in sea level, driven by the Indian Ocean Dipole, led to the mangrove dieback in the Maldives in 2020. Prolonged exposure to sea water from a rise in mean sea level meant mangroves experienced a greater influx of saline water, which likely created higher porewater salt concentrations^48^. Porewater salinity was likely exacerbated by increased evapotranspiration due to the high temperatures in 2020^19^. As salinity tolerances were exceeded, sediment accretion decreased, increasing soil submergence and further increasing salinity stress. We suggest this mechanism created a positive feedback loop that likely led to the observed tree mortality. Mangrove vulnerability was compounded by the geomorphic setting of the forests and the low salt-tolerance of the mangrove species (*B*. *cylindrica*).

While mangroves do go through natural die-off phases^20^, the extreme magnitude of dieback experienced in the Maldives combined with our multiple lines of evidence suggest this was possibly not a natural phase*.* As existing literature suggests that the frequency and magnitude of strong positive Indian Ocean Dipole events may increase climate change^54^, the re-occurance of these events in combination with relative sea-level rise, could threaten the stability of mangrove habitats in the Maldives and other Small Island Developing States in the Western Indian Ocean^14^. For these nations, annual average sea level was positively correlated with the Dipole Mode Index (Supplementary 7). During 2019 and 2020, mangrove area decreased by < 1% in the Seychelles (0.10 ha) and 1% Comoros (0.96 ha) which may have been caused by the same positive Indian Ocean Dipole event^45^. In addition to this dieback event, mangrove loss was observed between 2019 and 2020 on other islands within the Western Indian Ocean, such as a 1% decrease in Mayotte (6.5 ha) and < 1% in Madagascar (422.2 ha)^55^. Such considerable losses in mangrove forest area, across a large spatial scale, highlight the urgent need for increased awareness and understanding of how mangroves in the Western Indian Ocean respond to sea-level rise and the Indian Ocean Dipole. Investing in restoration projects is critical to increase mangrove and island physical resilience to the growing threat of climate change and extreme climatic events^7^.

## Methods

### Island selection

Islands were selected as local communities confirmed dieback on these islands and because forest mortality was readily visible in satellite imagery. To address variations in land use, we selected both uninhabited and inhabited islands and, to account for the different sea level and climate zones, we included islands from the north and south of the archipelago.

### Remote sensing

The satellite data used for this study were retrieved from the Landsat 8 OLI (30 m pixel resolution; collection 2, tier 1) surface reflectance image collection hosted in Google Earth Engine (GEE)^56^. An image selection protocol was developed whereby images with cloud cover of > 10% (measured using image property ‘CLOUD_COVER’) were discarded following a series of trials with varying cloud cover thresholds. Next, annual composite pseudo-images (*n* = 8 composites) were generated for six islands in the Maldives across an eight-year time series (2014 to 2022). Image compositing combines spatially overlapping images into a single image based on an aggregation function and is a robust data reduction technique that minimizes image artefacts^57^.

To track temporal trends in mangrove health through time, firstly, a supervised random forest machine learning classification was generated to map the most recent, 2022, extent of mangrove dieback. Training pixels for this classification were generated from photo interpretation of high-resolution imagery and supported by consultation with local experts. The area of dead mangrove from the random forest classification was then used as an area of interest to measure changes in Normalised Difference Vegetation Index (NDVI) values across previous years. For each year we recorded average, minimum, and maximum NDVI values. Considered a ‘greenness’ index, NDVI is a measure of photosynthetic activity of plants and is highly correlated to canopy closure in mangroves^19^. Thus, it has been used to monitor shifts in mangrove health through time in relation to human or climate disturbances^45^. NDVI measures the absorbance of the red band by chlorophyll and the reflection of the near-infrared by the mesophyll leaf structure^45^. Values range from − 1 to + 1, with values below zero not related to green vegetation. A higher NDVI value (i.e., closer to 1) is indicative of dense closed canopy forests whereas a lower value suggests sparse vegetation^19^. NDVI was calculated using the formula of Rouse et al.^58^:

$$NDVI = \frac{{\left( {NIR - R} \right)}}{{\left( {NIR + R} \right)}}$$

where *NIR* = the near infrared band and *R* = the red band.

### Dendrology and sediment geochemistry

#### Sampling

Mangrove wood and sediment samples were collected from GDh. Hoandedhdhoo in April and November 2022, approximately 24 to 28 months after the dieback event. Wood samples from *B. cylindrica* were collected from living (*n* = 9) and dead (*n* = 12) trees across three transects (Fig. 1E). Dead trees were situated in the middle of the dieback zone and living trees were on the edge of the mangrove forest. Samples were taken at breast height by cutting a 2 cm wood core from the trunk. A sediment core was taken to refusal (approximately 70 cm depth below the island surface) in the middle of the dieback area using a PVC pipe with an internal diameter of 4.25 cm (Fig. 1E). Sediment compaction was calculated (assuming uniform compaction) and accounted for in calculations.

### Mangrove wood analysis

Subsamples for *δ*^13^C analysis were taken from wood discs along the longest radius of each disc at regular intervals from the cores centre (oldest) to the outer edge (youngest)^18^. These subsamples were collected using a scalpel parallel to tree rings^18^. Alpha cellulose was extracted from the wooden subsamples, combusted to CO2 and measured for delta 13C using a Thermo-Fisher Delta V isotope ratio mass spectrometer at SUERC with an analytical precision of ± 0.1 (‰)^59^. Water use efficiency (WUE), the ratio of net photosynthesis to transpiration was calculated following Van Der Sleen et al.^60^.

### Radiometric dating and sediment accretion rates

The sediment core was sub-sectioned at 2.5 cm increments until 10 cm, then at 5 cm increments until the base of the core. Subsamples were dried and packed into petri dishes, then they were sealed and let to set aside for at least 21 days to stablish the secular equilibrium between ^210^Pb and its parents ^222^Rn and ^226^Ra. Sediment accretion rates were calculated by measuring excess ^210^Pb^61^. Given the half-life of ^210^Pb is 22.3 years, determination of sedimentation rate can span up to the last 100 years (approximately 5 times its half-life). Thus, the dating range of ^210^Pb makes it highly suitable for this analysis (< 50 years). Measurements and calculation were performed by the methods detailed in Breithaupt et al.^52^. In short, gamma activities were measured using an intrinsic germanium plain detector coupled to a multichannel analyser, and ^210^Pb specific activity was measured by the 46.5 keV peak and ^226^Ra by using its proxy ^214^Pb^52,61^. To establish the sediment chronologies, we used the Constant Rate and Supply (CRS) dating model, which was chosen due to the high sediment organic matter content of the core. This model assumes the constant ^210^Pb excess supply and allows sedimentation rate variation, that can be caused by dilution with both organic and inorganic sediments when sedimentation increases^62^. To calculate sediment accretion rates (mm/year), the depth of each interval was divided by the age variation (years). Sediment accretion rates were multiplied by the dry bulk density of each interval to calculate mass accumulation rates (g/cm^2^/year).

### Statistical information

The relationships between NDVI and year, as well as NDVI and sea level height (mm) were assessed using linear regression analyses implemented in R (version 4.4.0). We obtained sea level data from tide gauge monitoring stations at HDh. Hanimaadhoo (UHSLC ID: 108), Malé (UHSLC ID: 109) and S. Gan (UHSLC ID: 117) from the University of Hawaii Sea Level Centre^63^. To examine the relationship between sea level height in the Maldives and the Indian Ocean Dipole, the Dipole Mode Index was acquired for the Western Indian Ocean from NOAA^64^. We employed a paired *t-test* in R (version 4.4.0) to compare annual average NDVI before (*n* = 30) and after (*n* = 23) 2019 on all study islands and to examine the difference in *δ*^13^C and WUE between living (*n* = 9) and dead (*n* = 12) zones.

## Supplementary Information

Supplementary Information 1.

Supplementary Information 2.

## Data Availability

All data generated or analysed during this study are included in this published article [and its supplementary information files].

## References

[1] Ball, M. C. Ecophysiology of mangroves. *Trees* **2**, 129–142 (1988).

[2] Ruslan, N. F. N., Goh, H. C., Hattam, C., Edwards-Jones, A. & Moh, H. H. Mangrove ecosystem services: Contribution to the well-being of the coastal communities in Klang Islands. *Mar. Policy* **144**, 105222 (2022).

[3] Veitayaki, J., Waqalevu, V., Varea, R. & Rollings, N. *Mangroves in Small Island Development States in the Pacific: An Overview of a Highly Important and Seriously Threatened Resource*. (2017). 10.1007/978-4-431-56481-2_11

[4] Menéndez, P., Losada, I. J., Torres-Ortega, S., Narayan, S. & Beck, M. W. The global flood protection benefits of mangroves. *Sci. Rep.* **10**, 1–11 (2020). 32157114 10.1038/s41598-020-61136-6 PMC7064529

[5] IPCC. The Ocean and Cryosphere in a Changing Climate. (2019).

[6] Saintilan, N. *et al.* Thresholds of mangrove survival under rapid sea level rise. *Science.* **368**, 1118–1121 (2020). 32499441 10.1126/science.aba2656

[7] Saintilan, N. *et al.* Widespread retreat of coastal habitat is likely at warming levels above 1.5 °C. *Nature*10.1038/s41586-023-06448-z (2023). 37648850 10.1038/s41586-023-06448-z PMC10482694

[8] Lovelock, C. E. *et al.* The vulnerability of Indo-Pacific mangrove forests to sea-level rise. *Nature* **526**, 559–563 (2015). 26466567 10.1038/nature15538

[9] Krauss, K. W. *et al.* How mangrove forests adjust to rising sea level. *New Phytol.* **202**, 19–34 (2014). 24251960 10.1111/nph.12605

[10] Alongi, D. M. The impact of climate change on mangrove forests. *Curr. Clim. Chang. Reports* **1**, 30–39 (2015).

[11] Ellison, J. C. Mangrove retreat with rising sea-level, Bermuda. *Estuar. Coast. Shelf Sci.* **37**, 75–87 (1993).

[12] Cahoon, D. R. *et al.* Mass tree mortality leads to mangrove peat collapse at Bay Islands, Honduras after Hurricane Mitch. *J. Ecol.* **91**, 1093–1105 (2003).

[13] Chambers, L. G., Steinmuller, H. E. & Breithaupt, J. L. Toward a mechanistic understanding of “peat collapse” and its potential contribution to coastal wetland loss. *Ecology* **100**, 1–15 (2019). 10.1002/ecy.2720 PMC6850666 30933312

[14] Harris, R. M. B. *et al.* Biological responses to the press and pulse of climate trends and extreme events. *Nat. Clim. Chang.* **8**, 579–587 (2018).

[15] Abhik, S. *et al.* Influence of the 2015–2016 El Niño on the record-breaking mangrove dieback along northern Australia coast. *Sci. Rep.* **11**, 1–12 (2021). 34650104 10.1038/s41598-021-99313-w PMC8516887

[16] Duke, N. C. *et al.* ENSO-driven extreme oscillations in mean sea level destabilise critical shoreline mangroves—An emerging threat. *PLOS Clim.* **1**, e0000037 (2022).

[17] Duke, N. C. *et al.* Large-scale dieback of mangroves in Australia’s Gulf of Carpentaria: A severe ecosystem response, coincidental with an unusually extreme weather event. *Mar. Freshw. Res.* **68**, 1816–1829 (2017).

[18] Sippo, J. Z. *et al.* Reconstructing extreme climatic and geochemical conditions during the largest natural mangrove dieback on record. *Biogeosciences* **17**, 4707–4726 (2020).

[19] Lovelock, C. E., Feller, I. C., Reef, R., Hickey, S. & Ball, M. C. Mangrove dieback during fluctuating sea levels. *Sci. Rep.* **7**, 1–8 (2017). 28490782 10.1038/s41598-017-01927-6 PMC5431776

[20] Jimenez, J. A., Lugo, A. E. & Cintron, G. Tree Mortality in Mangrove Forests. *Biotropica* **17**, 177–185 (1985).

[21] Nurse, L. A. *et al.* Small Islands. *Clim. Chang. 2014 Impacts, Adapt. Vulnerability Part B Reg. Asp. Work. Gr. II Contrib. to Fifth Assess. Rep. Intergov. Panel Clim. Chang.* 1613–1654 (2014) 10.1017/CBO9781107415386.009.

[22] Crow, B. & Carney, J. Commercializing nature: mangrove conservation and female oyster collectors in The Gambia. *Antipode* **45**, 275–293 (2013).

[23] Beitl, C. M. Navigating over space and time: Fishing effort allocation and the development of customary norms in an open-access mangrove estuary in Ecuador. *Hum. Ecol.* **42**, 395–411 (2014).

[24] Zu Ermgassen, P. S. E. *et al.* Fishers who rely on mangroves: Modelling and mapping the global intensity of mangrove-associated fisheries. *Estuar. Coast. Shelf Sci.* **247**, 106975 (2020).

[25] UN-OHRLLS. 2015. Small IslandDeveloping States In Numbers: Climate Change Edition 2015.New York: UN-OHRLLS. http://unohrlls.org/custom-content/uploads/2015/12/SIDS-IN-NUMBERS-CLIMATE-CHANGE-EDITION_2015.pdf

[26] United Nations Conference on Trade and Development: List of Least Developed Countries. https://unctad.org/topic/least-developed-countries/list

[27] MEE. National Awareness Strategy for Water and Sewerage. (2017).

[28] Kench, P. S. *et al.* Climate-forced sea-level lowstands in the Indian Ocean during the last two millennia. *Nat. Geosci.* **13**, 61–64 (2020).

[29] Bailey, R. T., Khalil, A. & Chatikavanij, V. Estimating transient freshwater lens dynamics for atoll islands of the Maldives. *J. Hydrol.* **515**, 247–256 (2014).

[30] Sippo, J. Z., Lovelock, C. E., Santos, I. R., Sanders, C. J. & Maher, D. T. Mangrove mortality in a changing climate: An overview. *Estuar. Coast. Shelf Sci.* **215**, 241–249 (2018).

[31] Werner, A. D., Sharp, H. K., Galvis, S. C., Post, V. E. A. & Sinclair, P. Hydrogeology and management of freshwater lenses on atoll islands: Review of current knowledge and research needs. *J. Hydrol.* **551**, 819–844 (2017).

[32] Kench, P. S., Smithers, S. G., McLean, R. F. & Nichol, S. L. Holocene reef growth in the Maldives: Evidence of a mid-Holocene sea-level highstand in the central Indian Ocean. *Geology* **37**, 455–458 (2009).

[33] Worthington, T. A. *et al.* A global biophysical typology of mangroves and its relevance for ecosystem structure and deforestation. *Sci. Rep.* **10**, 1–11 (2020). 32887898 10.1038/s41598-020-71194-5 PMC7473852

[34] Cerri, F. *et al.* Mangroves of the Maldives: A review of their distribution, diversity, ecological importance and biodiversity of associated flora and fauna. *Aquat. Sci.* **86**, 44 (2024).

[35] Lagomasino, D. *et al.* Storm surge and ponding explain mangrove dieback in southwest Florida following Hurricane Irma. *Nat. Commun.*10.1038/s41467-021-24253-y (2021). 34183663 10.1038/s41467-021-24253-y PMC8238932

[36] Reef, R. & Lovelock, C. E. Regulation of water balance in Mangroves. *Ann. Bot.* **115**, 385–395 (2015). 25157072 10.1093/aob/mcu174 PMC4332601

[37] Shazra, A., Rasheed, S. & Ansari, A. Study on the mangrove ecosystem in Maldives. *Glob. J. Environ. Res.* **2**, 84–86 (2008).

[38] Maldives Meteorological Service: General Climate. https://www.meteorology.gov.mv/climate (2024)

[39] NASA: Sea Level Evaluation & Assessment Tool. https://sealevel.nasa.gov/data_tools/16 (2024)

[40] East, H. K., Perry, C. T., Kench, P. S., Liang, Y. & Gulliver, P. Coral reef island initiation and development under higher than present sea levels. *Geophys. Res. Lett.* **45**, 11265–11274 (2018).

[41] Saji, N. H., Goswami, B. N., Vinayachandran, P. N. & Yamagata, T. A dipole mode in the tropical Indian ocean. *Nature* **401**, 360–363 (1999). 16862108 10.1038/43854

[42] Wang, G., Cai, W., Yang, K., Santoso, A. & Yamagata, T. A unique feature of the 2019 extreme positive Indian Ocean Dipole event. *Geophys. Res. Lett.* **47**, 1–9 (2020).

[43] Manatsa, D., Chipindu, B. & Behera, S. K. Shifts in IOD and their impacts on association with East Africa rainfall. *Theor. Appl. Climatol.* **110**, 115–128 (2012).

[44] Foley, A. & Kelman, I. Precipitation responses to ENSO and IOD in the Maldives: Implications of large-scale modes of climate variability in weather-related preparedness. *Int. J. Disaster Risk Reduct.* **50**, 101726 (2020).

[45] Tran, T. V., Reef, R. & Zhu, X. A review of spectral indices for mangrove remote sensing. *Remote Sens.* **14**, 4868 (2022).

[46] Farquhar, G. D., Ball, M. C., von Caemmerer, S. & Roksandic, Z. Effect of salinity and humidity on δ13C value of halophytes-Evidence for diffusional isotope fractionation determined by the ratio of intercellular/atmospheric partial pressure of CO2 under different environmental conditions. *Oecologia* **52**, 121–124 (1982). 28310117 10.1007/BF00349020

[47] Farquhar, G. D., O’Leary, M. H. & Berry, J. A. On the relationship between carbon isotope discrimination and the intercellular carbon dioxide concentration in leaves. *Aust. J. Plant Physiol.* **9**, 121–137 (1982).

[48] Dittmann, S. *et al.* Effects of extreme salinity stress on a temperate mangrove ecosystem. *Front. For. Glob. Chang.* **5**, 1–18 (2022).

[49] Kirwan, M. L., Langley, J. A., Guntenspergen, G. R. & Megonigal, J. P. The impact of sea-level rise on organic matter decay rates in Chesapeake Bay brackish tidal marshes. *Biogeosciences* **10**, 1869–1876 (2013).

[50] Sasmito, S. D., Murdiyarso, D., Friess, D. A. & Kurnianto, S. Can mangroves keep pace with contemporary sea level rise? A global data review. *Wetl. Ecol. Manag.* **24**, 263–278 (2016).

[51] Breithaupt, J. L. *et al.* Partitioning the relative contributions of organic matter and mineral sediment to accretion rates in carbonate platform mangrove soils. *Mar. Geol.* **390**, 170–180 (2017).

[52] Breithaupt, J. L., Smoak, J. M., Smith, T. J. & Sanders, C. J. Temporal variability of carbon and nutrient burial, sediment accretion, and mass accumulation over the past century in a carbonate platform mangrove forest of the Florida Everglades. *J. Geophys. Res. Biogeosciences* **119**, 2032–2048 (2014).

[53] McKee, K. L. Biophysical controls on accretion and elevation change in Caribbean mangrove ecosystems. *Estuar. Coast. Shelf Sci.* **91**, 475–483 (2011).

[54] Sun, S., Fang, Y., Zu, Y., Liu, L. & Li, K. Increased occurrences of early Indian Ocean Dipole under global warming. *Sci. Adv.* **8**, 1–10 (2022). 10.1126/sciadv.add6025 PMC9683701 36417541

[55] Global Mangrove Watch: Net Change. https://www.globalmangrovewatch.org/ (2023)

[56] Gorelick, N. *et al.* Google Earth Engine: Planetary-scale geospatial analysis for everyone. *Remote Sens. Environ.* **202**, 18–27 (2017).

[57] White, J. C. *et al.* Pixel-based image compositing for large-area dense time series applications and science. *Can. J. Remote Sens.* **40**, 192–212 (2014).

[58] Rouse Jr JW, Haas RH, Deering DW, Schell JA, Harlan JC. Monitoring the vernal advancement and retrogradation (green wave effect) of natural vegetation (1974).

[59] Ascough, P. et al. 14C Measurement of Samples for Environmental Science Applications at the National Environmental Isotope Facility (NEIF) Radiocarbon Laboratory, SUERC, UK. *Radiocarbon.* **26**, 1–2 (2023).

[60] Van Der Sleen, P. *et al.* No growth stimulation of tropical trees by 150 years of CO2 fertilization but water-use efficiency increased. *Nat. Geosci.* **8**, 24–28 (2015).

[61] Appleby, P. G., Nolan, P. J., Oldfield, F., Richardson, N. & Higgitt, S. R. 210Pb dating of lake sediments and ombrotrophic peats by gamma essay. *Sci. Total Environ.* **69**, 157–177 (1988).

[62] Appleby, P. G. & Oldfield, F. The calculation of lead-210 dates assuming a constant rate of supply of unsupported 210Pb to the sediment. *Catena* **5**, 1–8 (1978).

[63] Caldwell, P. C., M. A. Merrifield, P. R. Thompson (2015), Sea level measured by tide gauges from global oceans — the Joint Archive for Sea Level holdings (NCEI Accession 0019568), Version 5.5, *NOAA National Centers for Environmental Information*, Dataset, 10.7289/V5V40S7W.

[64] NOAA Working Group on Surface Pressure: Dipole Mode Index [Internet] Available from: https://psl.noaa.gov/gcos_wgsp/Timeseries/DMI/

