# Supplementary material for: Sea-level rise and extreme Indian Ocean Dipole explain mangrove dieback in the Maldives
Source: Sci Rep. 2024 Nov 12;14:27012. doi: 10.1038/s41598-024-73776-z (PMC11557579; doi:10.1038/s41598-024-73776-z)
Supplement: Supplementary file 2 — Supplementary Information 2. [file 41598_2024_73776_MOESM2_ESM.docx]

**Supplementary Material**

Sea level rise and extreme Indian Ocean Dipole explain mangrove dieback in the Maldives.

*Carruthers et al.*

***Supplementary Figure 1*** *–* ***Temporal trends in maximum annual temperature (°C). (A)*** *HDh. Hanimaadhoo (the North); and* ***(B)*** *GDh. Kaadedhdhoo (the South). The relationship between maximum annual temperature and the Western Indian Ocean Dipole Mode Index (DMI) for* ***(C)*** *HDh. Hanimaadhoo (the North); and* ***(D)*** *GDh. Kaadedhdhoo (the South). Temperature data is provided by the Maldives Meteorological Service and Dipole Mode Index data is provided by NOAA.*

***Supplementary Figure 2*** *–* ***The relationship between sea surface temperature (°C) and the Western Indian Ocean Dipole Mode Index (DMI) in the Maldives****.. Sea surface temperature data and Dipole Mode Index data is provided by NOAA.*

***Supplementary Figure 3*** *–* ***Temporal trends in total annual precipitation (mm).*** ***(A)*** *HDh. Hanimaadhoo (the North); and* ***(B)*** *GDh. Kaadedhdhoo (the South). The relationship between in total annual precipitation and the Western Indian Ocean Dipole Mode Index (DMI) for* ***(C)*** *HDh. Hanimaadhoo (the North); and* ***(D)*** *GDh. Kaadedhdhoo (the South). Precipitation data is provided by the Maldives Meteorological Service and Dipole Mode Index data is provided by NOAA.*

| **Stat** | **Years** | | | | | | | | |
| --- | --- | --- | --- | --- | --- | --- | --- | --- | --- |
|  | **2014** | **2015** | **2016** | **2017** | **2018** | **2019** | **2020** | **2021** | **2022** |
| *Av* ± 1 S.D | 0.85 ± 0.03 | 0.86 ± 0.03 | 0.85 ± 0.05 | 0.85 ± 0.05 | 0.85 ± 0.05 | 0.83 ± 0.04 | 0.81 ± 0.07 | 0.70 ± 0.05 | 0.65 ± 0.05 |
| *Min* | 0.73 | 0.76 | 0.73 | 0.74 | 0.72 | 0.70 | 0.66 | 0.49 | 0.46 |
| *Max* | 0.91 | 0.91 | 0.77 | 0.91 | 0.92 | 0.90 | 0.90 | 0.85 | 0.84 |

***Supplementary Table 1*** *–* ***Summary table for NDVI of all study islands from 2014 to 2022.*** *Table includes average ± 1 S.D, minimum and maximum values.*

***Supplementary Figure 4*** *–* ***The relationship between annual average NDVI and annual mean sea level height (mm).*** ***(A)*** *islands in the north; and* ***(B)*** *GDh. Hoadedhdhoo (the South). The relationship between annual mean NDVI and annual mean sea level height (mm) with a one-year time lag for* ***(C)*** *the North; and (****D)*** *GDh. Hoadedhoo (the South).*

***Supplementary Figure 5*** *–* ***Temporal trends in NDVI from 2014 to 2022. (A)*** *Neykurendhoo,* ***(B)*** *Feydhoo,* ***(C)*** *Kelaa,* ***(D)*** *Goidhoo,* ***(E)*** *Keylakunu; and* ***(F)*** *Hoadedhdhoo (Imagery source, ESRI 2023).*

| **Depth interval**  **(cm)** | **Dry bulk density**  **(g/cm^3^)** | **Sediment accretion rate**  **(mm/year)** | **Mass accumulation rate**  **(g/cm^2^/year)** | **Age year**  **AD** |
| --- | --- | --- | --- | --- |
| 0 – 2.5 | 0.20 | 5.7 | 0.11 | 2020 |
| 2.5 – 5 | 0.09 | 7.1 | 0.06 | 2017 |
| 5 – 7.5 | 0.16 | 5.4 | 0.08 | 2010 |
| 7.5 - 10 | 0.14 | 3.7 | 0.05 | 1999 |
| 10 - 15 | 0.25 | 3.2 | 0.08 | 1983 |
| 15 - 20 | 0.27 | 2.8 | 0.08 | 1960 |

***Supplementary Table 2*** *–* ***Sediment core data****. Table includes dry bulk density (g/cm^3^), sediment accretion rate (mm/year), mass accumulation rate (g/cm^2^/year) and age of sample (Year AD).*

***Supplementary Figure 6*** *– Depth profile for (A) Total ^210^Pb activity (Bq/kg); and (B) Ra-226 (Bq/kg) activity.*

***Supplementary Figure 7*** *–****The relationship between sea level height (mm) and the Western Indian Ocean Dipole Mode Index (DMI) for other Small Island Developing States. (A)*** *Port Louis, Mauritius (UHSLC ID: 103)* ***(B)*** *Rodrigues, Mauritius (UHSLC ID: 105)* ***(C)*** *Pt. La Rue, Seychelles (UHSLC ID: 121); and* ***(D)*** *Diego Garcia (UHSLC ID: 104). Tide gauge data is provided by the University of Hawaii Sea Level Centre and Dipole Mode Index data is provided by NOAA.*

**Supplementary Methods**

**Mangrove wood**

***Figure 1*** *–Images of mangrove wood used for analysis in this study.*

The following was obtained from the Van Der Sleen et al. ^1^ method. δ^13^C_tree-ring_ (‰) was calculated as:

$\delta=\left[ \frac{R_{sample}}{R_{standard}}-1 \right] x 1000$	(1)

where $R_{sample}$ is the ^13^C/^12^C ratio of the sample and $R_{standard}$ is the ^13^C/^12^C of an internationally recognised standard material (V-PDB). Discrimination against the heavier ^13^C ($\triangle$^13^C) was calculated as:

$\triangle$^13^C = (δ^13^C_a_ - δ^13^C_tree-ring_) / (1 + δ^13^C_tree-ring_)	(2)

where δ^13^C_a_ is the δ^13^C of atmospheric CO_2_ (~8.1‰). Farquhar’s model was used for the discrimination of 13C in plants to estimate CO2 concentration in the intercellular spaces of the leaves (C_i_) from the discrimination values determined in equation 2:

$\triangle$^13^C **≈** a + (b – a) C_i_/C_a_	(3)

where a (4.4‰) is the slower diffusion of ^13^CO_2_ compared to ^12^CO_2_ through the stomata, b (27‰) is the discrimination by CO_2_ fixing enzyme Rubisco, C_a_ is the CO_2_ concentration of the atmosphere. Water use efficiency (WUE) was estimated from C_i_ as:

$WUE=\frac{A}{g_{s}}= \frac{\left( C_{a}{-C}_{i} \right)}{1.6}$	(4)

**^210^Pb dating**

***Figure 2*** *– Summary of sediment core including core photograph, lithology, unit description and ^210^Pb sampling location (with calculated dates).*

^210^Pb and ^226^Ra analysis was performed using an intrinsic germanium detector coupled to a multi-channel analyzer. Samples were sealed for at least 21 days to allow for ^222^Rn to reach secular equilibrium with ^226^Ra. ^210^Pb activity was determined by the direct measurement of the 46.5 KeV gamma peak while ^226^Ra activity was calculated averaging its daughters’ peaks ^214^Pb daughter and 214Bi (295.2 KeV) (351.9 KeV) (609.3 KeV) ^2^ Identical geometry was used for all samples. Activities were calculated by multiplying the counts per minute by a factor that includes the gamma-ray intensity and detector efficiency determined from standard calibrations ^3^. Unsupported (or excess) ^210^Pb activity (dpm/g) was calculated as:

$${Pb}_{unsupp}= {Pb}_{total}- {Pb}_{sup}$$

where Pb_unsupp_ is the unsupported (or excess) ^210^Pb activity, Pb_total_ is the total ^210^Pb activity and Pb_sup_ is the supported ^210^Pb activity.

**References**

1.	Van Der Sleen, P. *et al.* No growth stimulation of tropical trees by 150 years of CO2 fertilization but water-use efficiency increased. *Nat. Geosci.* **8**, 24–28 (2015).

2.	Appleby, P. G. & Oldfield, F. The calculation of lead-210 dates assuming a constant rate of supply of unsupported 210Pb to the sediment. *Catena* **5**, 1–8 (1978).

3.	Appleby, P. G., Nolan, P. J., Oldfield, F., Richardson, N. & Higgitt, S. R. 210Pb dating of lake sediments and ombrotrophic peats by gamma essay. *Sci. Total Environ.* **69**, 157–177 (1988).
